# Sodium–hydrogen exchanger NHA1 and NHA2 control sperm motility and male fertility

**DOI:** 10.1038/cddis.2016.65

**Published:** 2016-03-24

**Authors:** Su-Ren Chen, M Chen, S-L Deng, X-X Hao, X-X Wang, Y-X Liu

**Affiliations:** 1 State Key Laboratory of Stem Cell and Reproductive Biology, Institute of Zoology, Chinese Academy of Sciences, Beijing, China; 2 University of Chinese Academy of Sciences, Beijing, China

## Abstract

Our previous work identified NHA1, a testis-specific sodium–hydrogen exchanger, is specifically localized on the principal piece of mouse sperm flagellum. Our subsequent study suggested that the number of newborns and fertility rate of NHA1-vaccinated female mice are significantly stepped down. In order to define the physiological function of NHA1 in spermatozoa, we generated *Nha1*^Fx/Fx^, *Zp3*-Cre (hereafter called *Nha1* cKO) mice and found that *Nha1* cKO males were viable and subfertile with reduced sperm motility. Notably, cyclic AMP (cAMP) synthesis by soluble adenylyl cyclase (sAC) was attenuated in *Nha1* cKO spermatozoa and cAMP analogs restored sperm motility. Similar to *Nha1* cKO males, *Nha2*^Fx/Fx^, *Zp3*-Cre (hereafter called *Nha2* cKO) male mice were subfertile, indicating these two *Nha* genes may be functionally redundant. Furthermore, we demonstrated that male mice lacking *Nha1* and *Nha2* genes (hereafter called *Nha1/2* dKO mice) were completely infertile, with severely diminished sperm motility owing to attenuated sAC-cAMP signaling. Importantly, principal piece distribution of NHA1 in spermatozoa are phylogenetically conserved in spermatogenesis. Collectively, our data revealed that NHA1 and NHA2 function as a key sodium–hydrogen exchanger responsible for sperm motility after leaving the cauda epididymidis.

As many as 15% of human couples are infertile, and male infertility is about half of these cases.^1^ To fertilized egg, spermatozoa from the cauda epididymis must travel a long journey in the female reproductive tract to reach ampulla of uterine tube. Interestingly, in most mammalian species examined, the sperm journey experiences a natural increase in Na^+^/HCO_3_^−^ concentration and pH value (pH<7, Na^+^<25 mM, HCO_3_^−^<1 mM in cauda epididymis, whereas pH~7.4, Na^+^>100 mM, HCO_3_^−^>10 mM in female reproductive tract).^2, 3^ It is thus clear that intracellular pH (pHi) regulation is of the utmost importance for sperm physiology, including motility, maturation and the acrosome reaction.^4^ The maintenance of sperm pHi is kept through the involvement of several mechanisms, among which is included the sodium (Na^+^)–hydrogen (H^+^) exchangers (NHEs).^5^

NHEs, also known as Na^+^/H^+^ antiporters (NHAs), are integral membrane proteins that catalyze the exchange of Na^+^ for H^+^ across lipid bilayers and are ubiquitously distributed in almost all living organisms.^6^ The SLC9 gene family encodes NHEs and can be divided into three subgroups (reviewed in Martins *et al.*^7^). The SLC9A subgroup encompasses plasmalemmal isoforms NHE1–5 (SLC9A1–5) and the predominantly intracellular isoforms NHE6–9 (SLC9A6–9). The SLC9B subgroup consists of two recently cloned isoforms, NHA1 and NHA2 (SLC9B1 and SLC9B2, also known as NHEDC1 and NHEDC2). The SLC9C subgroup consist of a sperm-specific plasmalemmal NHE (SLC9C1, also known as sNHE) and a putative NHE, SLC9C2, for which there is currently no functional data.

Four Na^+^/H^+^ exchangers (NHE1,^8^ NHE5,^9^ sNHE^10^ and NHA1^11^) are reported to be expressed in spermatozoa. However, normal sperm motility is maintained in *Nhe1*-null mice, suggesting that *Nhe1* gene is male fertility independent.^12^ Testis histology, sperm numbers and morphology are normal, but *sNhe* null males are completely infertile with severely diminished sperm motility.^10^ Further study suggests that cyclic AMP (cAMP) metabolism is impaired in spermatozoa lacking sNHE.^13^ A recent study showed that NHE8 is highly expressed in the Leydig cells and male mice lacking *Nhe8* gene are infertile through its effect on modifying luteinizing hormone receptor (LHR) function.^14^

Second messenger cAMP has been reported to be essential for sperm function, including activation of motility, hyperactivation and acrosome reaction, mainly via activation of holoenzyme protein kinase A (PKA).^15^ In mammalian spermatozoa, cAMP is synthesized by a soluble isoform of the adenylyl cyclase (sAC) family.^16, 17^ There are two alternative splicing products, which independently encode full-length sAC (sAC_fl_) and truncated forms of sAC (sAC_t_).^18^ *sAC*-null male mice are infertile because of a severe defect in sperm motility.^19, 20^ In addition, as HCO_3_^−^ directly regulates sAC, this enzyme is able to translate pH changes into cAMP levels.^21, 22^

The sperm flagellum-specific NHE identified by Liu *et al.*^23^ in our laboratory in 2010 is now classified into a new family of NHE, NHA1 (SLC9B1, also known as NHEDC1). Our subsequent study demonstrates that anti-NHA1 antibody reduced sperm motility and the rate of *in vitro* fertilization.^23^ Therefore, NHA1 is proposed to regulate sperm motility. The critical role for NHA1 in human male fertility is highlighted by the finding that *NHA1* expression is either reduced or absent in patients with teratozoospermia.^24^

In order to define the physiological function of NHA1 in spermatozoa, we generated *Nha1* cKO, *Nha2* cKO and *Nha1/2* dKO male mice. Although single conditional knockouts for *Nha1* or *Nha2* were subfertile, male double knockout mice exhibited completely infertile with severely diminished sperm motility. cAMP synthesis by sAC was attenuated in cKO and dKO spermatozoa. Furthermore, the sperm motility defects could be rescued by the addition of cell-permeable cAMP analogs. In addition, the number of newborns and fertility rate of *Nha1/2*-vaccinated female mice were significantly stepped down, suggesting NHA1 and 2 may be an excellent target molecules for developing a novel male contraceptive.

## Results

### NHA1 and NHA2 expression in sperm

**Figure 1:**
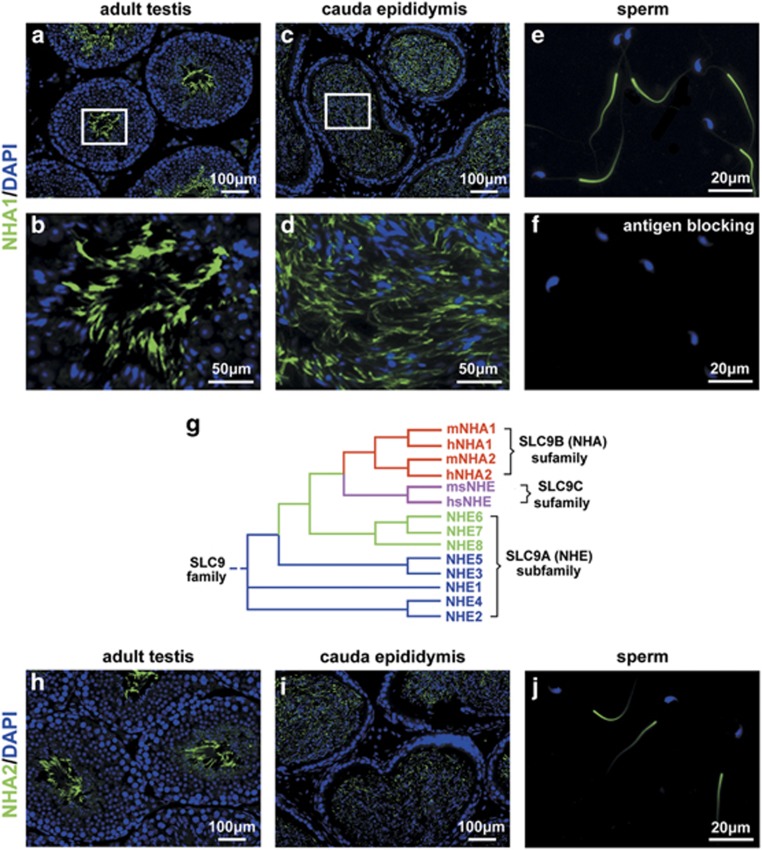
NHA1 and NHA2 were specifically expressed in the principal piece of sperm tail. (**a**-**f**) Immunofluorescence staining of NHA1 in mouse testis (**a** and **b**), cauda epididymis (**c** and **d**) and sperm from cauda epididymis (**e**). Note the intensive green signal at principal piece of sperm tail. NHA1 antibody staining in the presence of competing immunogen (**f**). The nuclei are counterstained with DAPI (blue). (**g**) Phylogenetic tree displays the relationship between the NHA1 and the other NHEs. The tree was generated with GeneBee aligning the predicted open reading frames of each NHE. (**h-j**) Immunofluorescence staining of NHA2 in mouse testis (**h**), cauda epididymis (**i**) and sperm from cauda epididymis (**j**). Scale bar in **a, c, h** and **i**, 100 *μ*m. Scale bar in **b** and **d**, 50 *μ*m. Scale bar in **e, f** and **j**, 20 *μ*m

Our previous reports suggest that NHA1 is exclusively expressed in mouse testis.^11, 23^ We further found that NHA1 was specifically localized to the sperm tail during late spermatogenesis (Figures 1a and b) and sperm storage in cauda epididymis (Figures 1c and d). High-magnification immunofluorescence of isolated sperm from cauda epididymis further demonstrated that the localization of NHA1 was confined to the principal piece of flagella (Figures 1e and f). *Nha1* seems most closely related to *Nha2* (Figure 1g). We further found that NHA2 is specifically localized in the principal piece of sperm tail, which is similar to NHA1 expression pattern (Figures 1h and j).

### Generation and analysis of *Nha1* knockout mice

**Figure 2:**
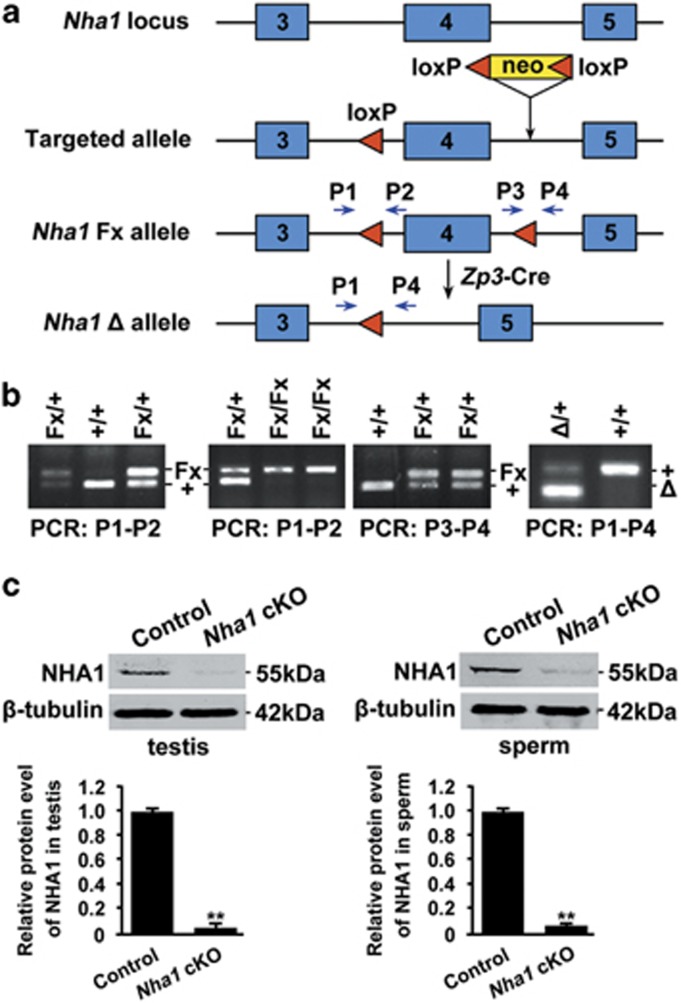
Diagram illustrates our targeting strategy and the creation of *Nha1* cKO mice. (**a**) In the targeted allele, a loxP site and a pgk-neo cassette flanked by two loxP sites are inserted into intron 3 and intron 4, respectively. Mice carrying the *Nha1*-targeted allele were crossed with the Cre transgenic mice to generate progeny carrying the *Nha1*Fx or *Nha1*△ allele. (**b**) PCR analysis detects the presence of 5' (PCR: P1–P2) and 3' (PCR: P3–P4) loxP sites for genotyping the wild-type (+/+), heterozygous (Fx/+) and homozygous (Fx/Fx) mice, and examines the deletion of exon 4 in the *Nha1*△/+ mice (PCR: P1–P4). (**c**) The knockout efficiency was confirmed by western blotting in testis and sperm samples. The protein level was normalized and plotted against *β*-tubulin. The data are expressed as the mean±S.D. **P<0.01

Our previous study suggests that polyclonal antibody to trans-membrane region of NHA1 significantly reduced the *in vitro* sperm motility and fertilization.^11^ To elucidate the physiological function of NHA1, we generated *Nha1* knockout mice by homologous recombination technology. LoxP sites flank exon 4 of the *Nha1* allele, and recombination of the loxP sites using Cre recombinase resulted in the removal of exon 4 (Figure 2a). The successful acquisition of *Nha1* cKO mice was determined by polymerase chain reaction (PCR) amplification (Figure 2b). We confirmed that NHA1 was successfully depleted in both testis and spermatozoa in *Nha1* cKO males (Figure 2c).

**Figure 3:**
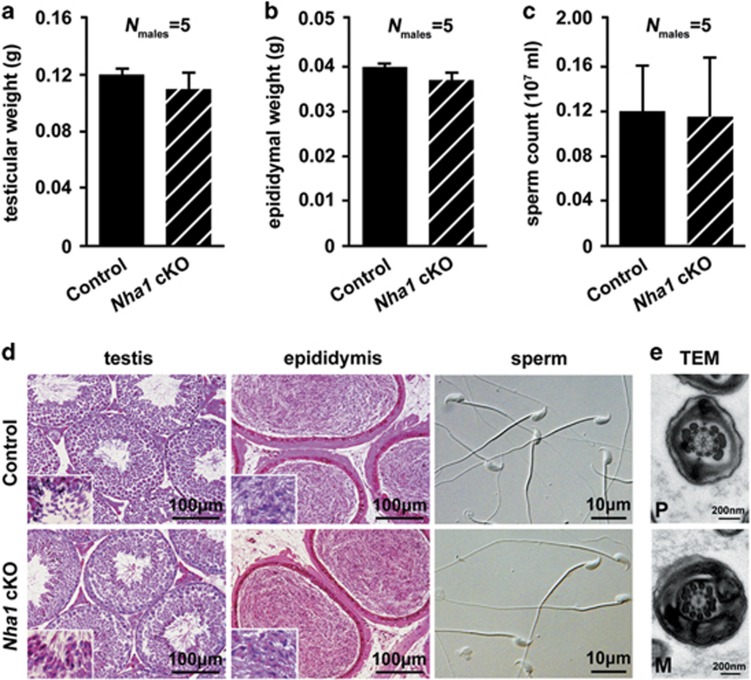
Testis and sperm morphology of *Nha1* cKO mice. (**a**-**c**) Testicular weight (**a**), epididymal weight (**b**) and sperm count (**c**) were examined. No overt abnormalities were found in the *Nha1* cKO mice. Data are expressed as the means±S.D. (*n*=5). (**d**) Testicular and cauda epididymal sections stained with H&E and spermatozoa obtained form cauda epididymis. No overt morphological abnormalities were observed in the testis or spermatozoa of *Nha1* cKO mice. (**e**) Sperm principal piece (*P*; upper panels) and midpiece (M; lower panels) were observed with TEM. No structural abnormalities were observed. Scale bar in **d** (the first two columns), 100 *μ*m. Scale bar in **d** (third column), 10 *μ*m. Scale bar in **e**, 200 nm

In *Nha1* cKO male mice, there were no overt abnormalities in testicular weight (123±4 mg in wild-type and 110±9 mg in cKO), epididymal weight (40±1 mg in wild-type and 36±2 mg in cKO) and sperm count (1.4±0.2 × 10^6^/ml in wild-type and 1.4±0.3 × 10^6^/ml in cKO) (Figures 3a and c). No overt abnormalities in spermatogenesis and epididymis of *Nha1* cKO mice (Figure 3d). Isolated sperm from cauda epididymis exhibited typical morphology (Figure 3d) and flagella structure (Figure 3e).

### NHA1 is essential for sperm motility and male fertility

**Figure 4:**
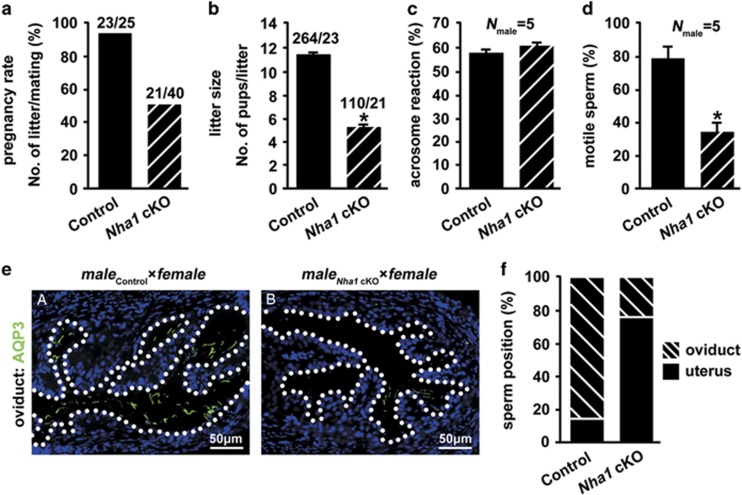
*Nha1* cKO male mice are subfertile. (**a**) Pregnancy rate was calculated as the ratio of the number of females with pregnancy to the number of females with successful mating. (**b**) When calculating average litter size, only the females that generated pups were included. (**c**) The percentage of sperm that underwent acrosome reaction (AR) after A23187 induction was counted. (**d**) Percentage motility of *Nha1* cKO and wild-type spermatozoa after release from the cauda epididymis. Data in **a–d** are expressed as the means±S.D., *P<0.05. (**e**) Oviduct was collected from females mated with *Nha1* knockout and wild-type males 4–6 h after coitus. Sperm tails were staining with AQP3 antibody (green). The nuclei are counterstained with DAPI (blue). Note that *Nha1* cKO sperm were seldomly observed in the oviduct. Scale bar in **d**, 50 *μ*m. (**f**) The sperm position was calculated in uterus or oviduct of females mated with *Nha1* cKO and wild-type males 4–6 h after coitus

By breeding assay, we found that although *Nha1* cKO males copulated, the pregnancy rate (92% in wild-type and 52.5% in cKO) and litter size (11.5±0.3 in wild-type and 5.2±0.4 in cKO) were markedly lower compared with their control littermates (Figures 4a and b). Acrosome reaction, a prerequisite for spermatozoa to fertilize eggs, occurred normally in *Nha1* cKO spermatozoa (acrosome rate: 58±1% in wild-type and 61±1% in cKO) (Figure 4c). However, percentage motility of *Nha1* cKO spermatozoa after release from the caudal epididymis was significantly lower than those of control spermatozoa (82±3% in wild-type and 38±2% in cKO) (Figure 4d). Moreover, fewer spermatozoa of *Nha1* cKO males reached the female oviduct 4–6 h after coitus, compared with those of controls (Figures 4e and f).

### cAMP analogs rescue the motility defects of *Nha1* cKO spermatozoa

**Figure 5:**
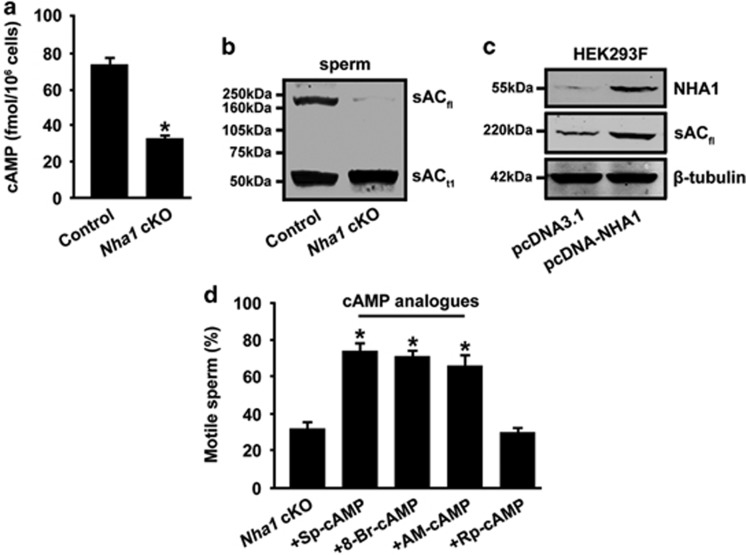
sAC-mediated cAMP signaling is compromised in *Nha1* cKO spermatozoa. (**a**) Sperm from wild-type and *Nha1* cKO mice were collected in a bicarbonate-free medium. cAMP content was measured by using RIA. Data represent the mean±S.D. (*n*=3). (**b**) Percentage motility of null spermatozoa after a 60-min incubation with cAMP analogs plus 100 *μ*M IBMX. Sp-cAMP (1 mM); 8-Br-cAMP (1 mM); AM: AM-cAMP (50 μM); Rp-cAMP is the biologically inactive cAMP analog (1 mM). Data in **a** and **b** are expressed as the means±S.D., *P<0.05. (**c**) Immunoblot of total sperm cell lysate (2 × 10^6^ cells) from wild-type or *Nha1* cKO mice with anti-sAC antibody, which detected two major proteins: a sAC_fl_ and a truncated sAC (sAC_t1_). (**d**) HEK293F cells were trasfected with pcDNA-*Nha1* or empty pcDNA3.1 vector. Total cell lysates were used for immunoblotting with anti-NHA1 or anti-sAC antibody. The protein level was normalized and plotted against *β*-tubulin

Our previous study suggests that sperm pHi descends after treatment with the NHA1 antisera.^11^ Given that cAMP synthesis by sAC is closely related to sperm pHi regulation and sperm motility, we detected the levels of cAMP and sAC in *Nha1* cKO spermatozoa. Basal cAMP concentrations were significantly lower in *Nha1* cKO spermatozoa compared with wild-type sperm cells (76±3 fmol/10^6^ cells in wild-type and 34±2 fmol/10^6^ cells in cKO) (Figure 5a). The protein level of sAC_fl_, a cAMP synthetase, was greatly diminished in *Nha1* cKO spermatozoa (Figure 5b). Moreover, transfection of NHA1 enhanced sAC_fl_ protein expression in HEK293F cells (Figure 5c). Notably, addition of membrane-permeable cAMP analogs, such as Sp-cAMP, 8-Br-cAMP and AM-cAMP, almost fully recovered the sperm motility of *Nha1* cKO sperm (Figure 5d).

### Generation of *Nha2* cKO and *Nha1/2* dKO mice

Nucleotide sequence is very similar between *Nha1* and *Nha2*, indicating two *Nha* genes may be functionally redundant. Accordingly, we generated *Nha2* cKO and *Nha1/2* dKO mice. LoxP sites flank exon 3 of the *Nha2* allele, and recombination of the loxP sites using Cre recombinase resulted in the removal of exon 4 (Supplementary Figures S1a-c). Afterward, we generated *Nha1/2* dKO mice (Supplementary Figure S1d).

Similar to *Nha1* cKO, no overt abnormalities in testicular weight (120±3 mg in wild-type and 112±4 mg in cKO), epididymal weight (38±1 mg in wild-type and 36±2 mg in cKO) and sperm count (1.3±0.2 × 10^6^/ml in wild-type and 1.3±0.3 × 10^6^/ml in cKO), spermatogenesis, epididymis and epididymal sperm structure of *Nha2* cKO males (Supplementary Figures S2a-f). The pregnancy rate (91.6% in wild-type and 65% in cKO) and litter size (11.5±0.2 in wild-type and 7.2±0.3 in cKO) of *Nha2* cKO males were markedly lower compared with their control littermates (Supplementary Figures S3a and b). *Nha2* deletion significantly reduced the percentage of motile sperm (84±2% in wild-type and 56±3% in cKO) (Supplementary Figure S3c) and the cAMP synthesis by sAC (Supplementary Figures S3d and e).

In addition, the mRNA level of *Nha2* was significantly increased in the *Nha1* cKO sperm (Supplementary Figure S4a). Similarly, significant upregulation of *Nha1* level was observed after deletion of *Nha2* (Supplementary Figure S4b). Collectively, these two *Nha* genes are functionally redundant.

### *Nha1/2* dKO males are infertile

No obvious behavioral and gross anatomical differences were noted when comparing adult *Nha1/2* dKO males and wild-type littermates. Also, no relevant changes in testicular weight (110±4 mg in wild-type and 104±6 mg in dKO), epididymal weight (37±1 mg in wid-type and 36±2 mg in dKO) and sperm count (1.3±0.2 × 10^6^/ml in wild-type and 1.2±0.2 × 10^6^/ml in dKO) were observed in *Nha1/2* dKO males (Supplementary Figures S2g-i). Elongated spermatids were present in testes and spermatozoa were present in the epididymis (Supplementary Figures S2j-k). Moreover, isolated sperm from cauda epididymis exhibited typical morphology (Supplementary Figure S2l).

**Figure 6:**
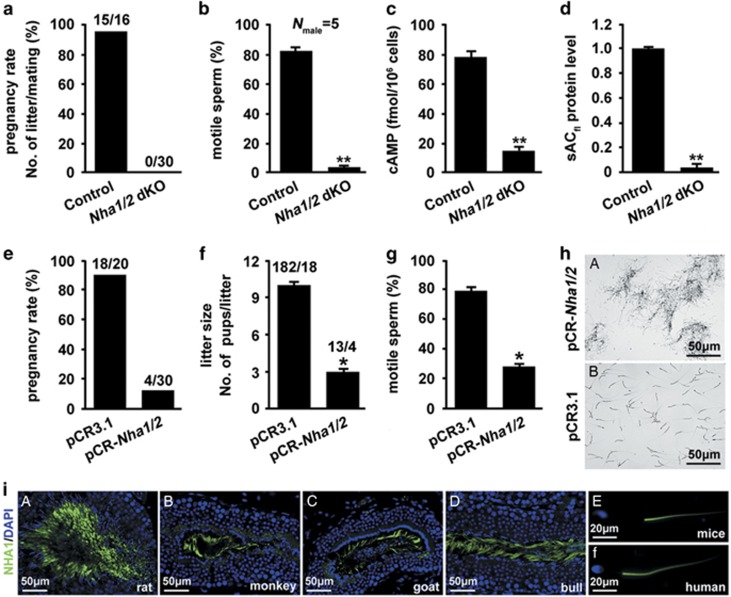
*Nha1/2* dKO males are infertile and potential contraceptive role of pCR-*Nha1/2* DNA vaccine. (**a**) Pregnancy rate is presented as a percentage. (**b**) Percentage motility of *Nha1/2* dKO and wild-type spermatozoa after release from the caudal epididymis. (**c**) cAMP content of spermatozoa was measured by using RIA. (**d**) The protein level of sAC_fl_ (normalized and plotted against *β*-tubulin) in *Nha1/2* dKO and wild-type spermatozoa. (**e** and **f**) The effect of the pCR-*Nha1/2* DNA vaccine on pregnancy rate (**e**) and litter size were examined (**f**). (**g**) Spermatozoa motility treated with the vaginal fluid of pCR-*Nha1/2* orally immunized mouse as compared with that of the pCR3.1 group. Data in **a–g** are expressed as the means±S.D., *P<0.05; **P<0.01. (**h**) Sperm suspensions of normal mouse were mixed with vaginal fluid of pCR-*Nha1/2* or pCR3.1 immunized females. Note that agglutination in a tangled pattern was only observed in pCR-*Nha1/2* group. Scale bar, 50 *μ*m. (**i**) Immunolocalization of NHA1 in tissue sections of testes or sperm sample from diverse mammalian species, including rat, monkey, goat, bull, mice and human. Scale bar in a–d, 50 *μ*m. Scale bar in e and f, 10 *μ*m

Mating behavior of male *Nha1/2* dKO mice was normal because vaginal plugs were observed in the females. However, mating of *Nha1/2* dKO male mice with wild-type females for 2 months did not produce any pregnancies (Figure 6a). Motility analysis indicated that sperm motility was drastically reduced in *Nha1/2* dKO males when compared with control littermates (84±2% in wild-type and 5±1% in dKO) (Figure 6b). *Nha1/2* dKO sperm produced only a slightly trembling movement of the flagella. Significant reduced levels of cAMP (80±2 fmol/10^6^ cells in wild-type and 17±2 fmol/10^6^ cells in dKO) and sAC_fl_ (<20-fold) were observed in *Nha1/2* dKO sperm cells (Figures 6c and d).

### Contraceptive function of pCR-*Nha1/2* vaccine

Oral vaccination of female mice with pCR-*Nha1/2* significantly reduced both the pregnancy rate (90% in control and 13% in vaccinated group) and the number of newborns (10.3±0.2 in control and 3.2±0.2 in vaccinated group), as compared with the pCR3.1 mock plasmid (Figures 6e and f). Sperm motility was statistically reduced when sperm were mixed with vaginal wash from the pCR-*Nha1/2* orally vaccinated females (80±2% in control and 27±1% in vaccinated group) (Figure 6g). Notably, sperm agglutination was only observed as the cross-linking or clumping together when sperm were treated with vaginal wash from the pCR-*Nha1/*2-vaccinated female mice (Figure 6h).

### Sperm distribution of NHA1 are present across mammalian species

To determine whether sperm distribution of NHA1 is phylogenetically conserved in spermatogenesis, we performed immunolocalization of NHA1 in testes or sperm from diverse mammalian species, including rat, monkey, goat, bull, mouse and human (Figure 6i). As expected, NHA1 was specifically localized to the sperm tail during late spermatogenesis and confined to the principal piece of flagella.

## Discussion

Fertilization is a fundamental and convoluted process. However, we are still far from fully understanding the gamete intracellular molecular dialog necessary for fertilization. Furthermore, it is needed to tackle increasing male infertility and to provide safer male gamete-based contraceptives. Pathological and pharmacological studies of the specific ion channel from spermatozoa, in the species where it can be done (i.e., null mice), will yield clear evidence of the role of the channel in sperm physiology and final fertilization. In this study, we showed that sodium–hydrogen exchangers, NHA1 and NHA2, were essential for sperm function as their elimination caused infertility in male mice because of a severe defect on sperm motility.

Several unique sperm ion transporters and enzymes whose elimination causes infertility are either pHi dependent or somehow related to pHi regulation. Among them are: CatSper, a Ca^2+^ channel; Slo3, a K^+^ channel; Na^+^/H^+^ exchanger and the sAC (reviewed in Nishigaki *et al.*^4^). An increase in bicarbonate (HCO_3_^−^) during the transit from the epididymis to the female genital tract (HCO_3_^−^<1 mM in cauda epididymis, whereas HCO_3_^−^>10 mM in female reproductive tract) activates sAC.^19, 20, 25^ HCO_3_^−^-induced cAMP synthesis by sAC stimulates PKA and promotes sperm maturation, motility and capacitation in the female genital tract.^26, 27, 28^ In addition to PKA, cAMP is able to bind and regulate other proteins containing cyclic nucleotide binding domain (CNBD).^29^ A CNBD protein known to be present in mouse sperm is a specific member of the mammalian NHE superfamily of Na^+^/H^+^ exchangers, sNHE.^10^ Targeted deletion of *sNhe* gene results in infertility because of severely diminished sperm motility. Interestingly, sperm from *sNhe* null mice lack motility but either permeable cAMP analogs or NH_4_Cl rescues this phenotype.^10^ Later report from the same group showed that sNHE is necessary for the full-length expression of sAC suggesting that these two proteins form a protein complex involved in sperm pHi control.^13^ Thus, it is proposed that cAMP affects the sperm pHi by controlling the sNHE through its CNBD.^30^

The phenotype of *Nha1* cKO, *Nha2* cKO and *Nha1/2* dKO male mice is somewhat similar with *sNhe* null males as follows. First, male (sub)infertility is caused by severely diminished sperm motility. Second, their cAMP synthesis by sAC is highly diminished. Third, addition of membrane-permeable cAMP analogs almost fully recovered the sperm motility of null sperm. Quite distinct from NHA1 and NHA2, sNHE contains a consensus sequence for a putative CNBD near the C terminus, implying that sNHE function could be regulated by cyclic nucleotides. However, we did not observe any CNBD in the nucleotide sequences of *Nha1* or *Nha2* gene.

Based on knockout evidence, together with previous *in vitro* studies, we can infer that NHA1 and NHA2 are functional Na^+^/H^+^ exchangers, and thus, *Nha1* and *Nha2* deficiency causes sperm pHi reduction and immotility most likely via attenuating sAC-mediated cAMP synthesis. However, how NHA1 and NHA2 regulate sAC and the exact correlation between pHi and cAMP level are still unclear. Further experiments, such as proteomic analysis and co-immunoprecipitation, will be useful to reveal whether NHA1 and/or NHA2 are the sAC-associated proteins.

Besides, the defect of the *Nha1*-null sperm is clinically related, because *NHA1* expression is either reduced or absent in patients with teratozoospermia, a male infertility condition characterized by the presence of abnormally shaped sperm in the semen (microarray data; Pubmed: GDS2697/1555142_at/*SLC9B1*). Understanding the mechanisms regulating the expression of *NHA1* gene is also of great importance. A recent study by Kumar *et al.*^24^ identifies and characterizes regulator DNA elements in the 5' end of the human *NHA1* gene and suggests that DNA methylation at these elements can regulate its expression.

Previous studies identify several genes, which could be served as the candidate contraceptive targets, given their sperm-specific expression and absolute requirement for fertility.^10, 31, 32, 33^ We are currently suggesting that male mice lacking *Nha1* and *Nha2* genes, sperm Na^+^/H^+^ exchangers, are infertile, with seriously reduced sperm motility owing to attenuated sAC-mediated cAMP synthesis. Our study not only unveils a genetic basis for the role of Na^+^/H^+^ exchangers in sperm motility, but also provides an attractive contraceptive targets in humans. Given that NHA1 and NHA2 exhibit a high degree of homology among different species, *Nha1/2* DNA vaccine may be a good strategy for developing an immunocontraceptive vaccine for human and animal use. As shown in this study, the number of newborns and fertility rate of *Nha1/2*-vaccinated female mice were significantly stepped down. However, the contraceptive efficiency of orally delivered *Nha1/2* DNA vaccine remains to be improved.

## Materials and Methods

### Ethics statement

All animal procedures were in accordance with the Animal Care and Use Committee (IACUC) of Institute of Zoology, Chinese Academy of Sciences.

### Antibody production

We produced anti-NHA1 polyclonal antisera obtained from the immunized rabbits using the purified NHA1 recombinant protein according to our previous study.^11^ Briefly, the selected partial cDNA sequence (encoding 62 amino acids) of the NHA1 was inserted into pGEX-4T-1 (Invitrogen, Carlsbad, CA, USA). The primer pair for cloning was: forward/*Bam*HI (5′-TTGGATCCCATGGATCTGGAGGACT-3′) and reverse/*Sal*I (5′-AAGTCGACGGCACATGCCAATGGTT-3′). The preparation of recombinant protein (GST plus NHA1 fragment) and immunization of rabbits were performed as describes.^34^ Six rabbits of each group were immunized and the antisera were harvested from arteriae carotis. We produced anti-NHA2 polyclonal antisera according to the above method. The primer pair for cloning (encoding 83 amino acids) of NHA2 was: forward/*Bam*HI (5′-TTGGATCCATCTACAGTTTTTAACATC-3′) and reverse/*Sal*I (5′-AAGTCGACTTGT CAGTCCACCTCATGCCTG-3′).

### Generation of *Nha1 and Nha2* knockout mouse model

A targeting vector containing *Nha1* exon 4 or *Nha2* exon 3 flanked by a loxP site and a loxP-Neo cassette was constructed and introduced into mouse embryonic stem (ES) cells (AB1, 129/SvEv) by electroporation. Next, ES cell clones containing the targeted *Nha1* or *Nha2* construct were injected into C57BL/6 (B6) blastocysts to generate chimeras. The resultant male chimeras were identified by coat color and mated with wild-type females to obtain germline transmission. Tail biopsies of agouti-pigmented F1 animals were genotyped using a primer set specific to the neo cassette. *Nha1*^+/Fx^ mice were mated with mice carrying *Nha1*^+/Fx^ and *Zp3*-Cre transgene mice (Cre recombinase expressed in the developing oocyte and in each cell of the resulting embryos) ^35^ to generate *Nha1* knockout mice. We used similar mating strategy to obtain *Nha2* knockout mice. The DNA isolated from tail biopsies was used for genotyping. The presence of the Fx allele and Cre transgene was determined by PCR amplification. Mice were genotyped by PCR analysis using primers (5′-CCAGCATTCTCACAAACC-3′ and 5′-AGACTGAACAAGCGAGCC-3′) to identify the loxP locus, primers (5′-TTTTCTCATTTTCCATTCAACTACA-3′ and 5′-AGCCCTACAATCCAGCCTTCAGAG-3′) to identify the exon 4 deleted locus for *Nha1* targeting. Mice were genotyped by PCR analysis using primers (5′-TTTTGTA GGCTCTTTTGC-3′ and 5′-CACAGGGGTTGAGTCATA-3′) to identify the loxP locus, primers (5′-TCGGAAGA GGGATACGGAAGT-3′ and 5′-ATTGGCCTGGATGAAAACGAG -3′) to identify the exon 3 deleted locus for *Nha2* targeting.

### Breeding assays

Eight to 12 weeks *Nha1* cKO (*n*=10), *Nha2* cKO (*n*=5), *Nha1/2* dKO (*n*=5) males and their control littermates males (*n*=5 each) were used for the breeding assay. Each male mouse was caged with two adult wild-type females (7–8 weeks) that were checked for vaginal plugs every morning. Once a vaginal plug was identified, another female was placed in the cage for another round of mating. The plugged female was separated and single caged, and the pregnancy results were recorded.

### Sperm count and motility assays

The cauda epididymis from 3-month-old male mice were placed in 1 ml modified HTF media and backflushing was used to obtain spermatozoa.^36^ Spermatozoa were incubated with DAPI (1 : 1000, Sigma-Aldrich, St. Louis, MO, USA) for 2 min. Samples were placed in Leja counting chambers (Orange Medical, Rotterdam, The Netherlands), and sperm concentration was evaluated by using the IVOS sperm analyzer (Hamilton Thorne Bioscience, St. Louis, MO, USA). Cauda epididymal spermatozoa in the media were treated with or without 50 *μ*M to 1 mM cAMP analogs, and 100 *μ*M IBMX (all purchased from Selleck, Shanghai, China). Motility of knockout spermatozoa after a 0- to 60-min incubation were analyzed by computer-assisted sperm analysis using the IVOS sperm analyzer (Hamilton Thorne Bioscience) and the motility rate (%) was measured.

### Hematoxylin and eosin (H&E) staining

Testes and cauda epididymis were fixed in Bouin's solution for 24 h. Following dehydration through an ethanol series, the fixed testes were embedded in paraffin and then sectioned. Paraffin sections (5-*μ*m-thick) were stained with H&E (Zhong Shan Technology, Beijing, China). Staining were examined under light microscopy (Nikon DS-Ri1, Tokyo, Japan).

### Immunofluorescence on tissue sections and epididymal sperm

Testes, cauda epididymis and oviduct were fixed in 4% paraformaldehyde (PFA) for 24 h, dehydrated and embedded in paraffin. The sections (5-*μ*m-thick) were deparaffinized and rehydrated; heat-induced antigen retrieval was performed in 10 mM sodium citrate buffer. Mouse sperm were collected from cauda epididymides using the backflushing method, washed in PBS, fixed in 4% PFA for 30 min at room temperature and air dried on poly-l-lysine-treated slides. Sections were blocked using a blocking buffer (5% donkey serum, 0.3% Triton X-100 in PBS) and incubated with primary antibodies against NHA1 (1 : 200, our laboratory) or NHA2 (1 : 200, our laboratory) or AQP3 (1 : 400, kind gift from Dr. Qi Chen) overnight at 4°C. Sections were washed and incubated with anti-rabbit FITC-conjugated secondary antibody (1 : 200, Jackson Immuno Research, West Grove, PA, USA) for 1 h and counterstained with DAPI. Images were captured using a fluorescence microscope (Nikon Eclipse 80i).

### Western blotting

Western blot analysis was performed as described previously.^37^ Briefly, testes and sperm were lysed in a radioimmune precipitation assay lysis buffer. The proteins were electrophoresed under reducing conditions on 10% SDS-PAGE gels and transferred to nitrocellulose membranes. The blots were incubated with the primary antibody overnight at 4 °C and then with the secondary antibody (anti-rabbit Dye 800CW, LI-COR, St. Louis, MO, USA) for 1 h at room temperature. The specific signals and the corresponding band intensities were evaluated using an Odyssey Infrared Imaging system (Odyssey, Berlin, Germany). The protein level was normalized and plotted against *β*-tubulin. The following antibodies were used in this study: rabbit anti-NHA1 (1/2000, our laboratory), rabbit anti-NHA2 (1/2000, our laboratory), rabbit anti-sAC (1/2000, Abgent, Cambridge, UK, AP5862c) and rabbit anti-*β*-tubulin (1/3000, Abcam, Cambridge, UK, 6046).

### Quantitative RT-PCR

RNA was extracted using Trizol (Invitrogen, Dallas, TX, USA) according to the manufacturer's protocol. RNA samples were subjected to reverse transcription using a PrimeScript RT Reagent Kit (Takara, Dalian, China). The reactions were run in triplicate in three independent experiments. The CT values for the samples were normalized to the corresponding *Gapdh* CT values, and relative expression levels were calculated using the ΔΔCT method. The primer pair for *Nha1*: forward (5′-TGAGCACGACGTAGAATCAAAC-3′) and reverse (5′-CAGGATCTTTGGACATCTCAACA-3′). The primer pair for *Nha2*: forward (5′-GCGAGCCTTTCTGGTTCTG-3′) and reverse (5′-CACCTCATGCCTGCTAGGA-3′).

### Immunization with Nha1/2 DNA vaccine

The purified PCR product was inserted into pCR3.1 (Invitrogen, San Diego, CA, USA) according to the manufacturer's instruction. The primer pair for cloning *Nha1* is: forward/*Hind*III (5′-GGCGAAGCTTGTTATGGGAGTTTTTG-3′) and reverse/*Eco*RI (5′-GCGGAATTCTTAATGATGGAAGTTCGAG-3′). The primer pair for cloning *Nha2* is: forward/*Hind*III (5′-GGCGAAGCTTCATGGTTGTTCTTCTG-3′) and reverse/*Eco*RI (5′-GCGGAATTCCAGAAATTTCTATGCTTC-3′). The female mice were immunized with the plasmid DNAs purified by using Qiagen Endofree Mega (Qiagen, St. Louis, MO, USA). Female mice were immunized with 20 *μ*g of *Nha1/2* vaccine or pCR3.1 mock plasmid dissolved in 30 *μ*l of saline via oral feeding. One week after the second immunization, two immunized groups (female mice) were paired with wild-type males for 3 month. They were checked daily for mating as evidenced by a vaginal plug. The number of female mice that gave birth was recorded.

### Sperm agglutination assay

One week after the second immunization, vaginal washes were collected. Sperm suspension (diluted to 20 × 10^6^ cells/ml) and vaginal fluid were mixed in a proportion of 3:1 (v/v) in a microcentrifuge tube. After incubation at 37 °C for 1 h, 50 *μ*l of the mixture was dropped on glass slides, and the sperm agglutination and motility were examined under light microscopy (Nikon DS-Ri1).

### Acrosome reaction

The A23187-induced acrosome reaction was analyzed as previously reported.^38^ Briefly, sperm were collected from the upper portion of HTF medium, and the calcium ionophore A23187 (Sigma) was added (final concentration 10 *μ*M) to induce the acrosome reaction. Fifteen minutes later, the sperm were spotted on a glass microscope slide, dried at room temperature and fixed with methanol for 30 s. Intact acrosomes were stained with FITC-PNA, and the sperm nuclei were labeled with DAPI. Sperm that had undergone the acrosome reaction could not be labeled with FITC-PNA. More than 200 sperm were examined for all experimental conditions.

### Transmission electronic microscopy (TEM)

Cauda epididymis was collected from wild-type and *Nha1* cKO mice and fixed overnight in 2.5% glutaraldehyde in 0.1 M phosphate buffer (pH 7.4), to allow the release of sperm. The sperm were collected and subsequently fixed in 2.5% (w/v) glutaraldehyde solution in phosphate buffer (pH 7.4) at 4 °C for 3 h. Then, the sperm were washed in phosphate buffer, collected on poly-l-lysine-coated glass coverslips, postfixed with 1.0% osmium tetroxide for 1 h, dehydrated in a graded series of ethanol and embedded in EPON/Araldite resin. Thin sections were cut, mounted on 200-mesh grids, and stained with uranyl acetate and lead citrate. The samples were then examined with a JSM-6360 LV scanning electron microscope (JEOL, Tokyo, Japan).

### cAMP RIA

Intracytoplasmic concentration of cAMP in sperm was determined using an RIA kit (Pasteur, Lyon, France), with ^125^I-labeled cAMP used as a tracer. Cauda epididymal spermatozoa (2 × 10^6^ cells) were released at 37 °C. The samples were first deproteinized by 0.4 N perchloric acid and then neutralized with 4 M potassic acetate for 24 h at 4 °C as described.^39^ Samples were then centrifuged at 800 *g* for 10 min at 4 °C. In all, 100 *μ*l of the supernatant was used for RIA performed, in duplicate, according to the manufacturer's directions.

### Cell transfection

HEK293F cells were grown in DMEM/F12 supplemented with 10% FBS at 37 °C with 5% CO_2_. The cells were seeded at a density of approximately 50 000 cells per well in 12-well plates 12 h before transfection. Cells were transfected with pcDNA-*Nha1* plasmid or empty pcDNA3.1 vector (Invitrogen, USA) via Lipofectamine 2000 (Invitrogen, USA) according to the manufacturer's recommendations. The primer pair for pcDNA-*Nha1* cloning is: forward (5′-TTTTAAAGTTCCCTGCTGAAACGTAAG-3′) and reverse (5′-GGTGATGATGGCAAGCTTTTTAATGAT-3′). Transiently transfected cells were harvested 48 h later for analysis.

### Statistical analysis

Data are expressed as the means±S.D. Statistical analyses were performed via ANOVA followed by Duncan's multiple range test for pairwise comparisons. Data analyses were performed using the SPSS software (SPSS Inc., Chicago, IL, USA). *P<0.05; **P<0.01.

## Abbreviations

pHi: intracellular pH
cAMP: cyclic AMP
sAC: soluble adenylyl cyclase
NHEs: sodium (Na^+^)–hydrogen (H^+^) exchangers
NHAs: Na^+^/H^+^ antiporters
LHR: luteinizing hormone receptor
PKA: protein kinase A
sAC_fl_: full-length sAC
sAC_t_: truncated forms of sAC
PCR: polymerase chain reaction
CNBD: cyclic nucleotide binding domain
ES: embryonic stem
H&E: hematoxylin and eosin
PFA: paraformaldehyde
TEM: transmission electronic microscopy
