# Supplementary material for: Sodium–hydrogen exchanger NHA1 and NHA2 control sperm motility and male fertility
Source: Cell Death Dis. 2016 Mar 24;7(3):e2152–. doi: 10.1038/cddis.2016.65 (PMC4823964; doi:10.1038/cddis.2016.65)
Supplement: Supplementary Figure Legends [file cddis201665x1.doc]

**Figure S1** Generation of *Nha2* cKO and *Nha1/2* dKO mice. (**a**) Schematic representation of the gene targeting in the *Nha2* locus by homologous recombination. Exon3 was deleted with Cre recombinase. (**b**) PCR analysis detects Fx and △. (**c**) The knockout efficiency was confirmed by western bloting in testis and sperm samples. (**d**) The knockout efficiency of NHA1/2 was confirmed by Western bloting using antibodies in sperm samples. The protein level was normalized and plotted against β-tubulin.

**Figure S2** Characteristics of *Nha2* cKO and *Nha1/2* dKO testes and sperm. (**a**-**c**) Testicular weight (a), epididymal weight (b) and sperm count (c) were examined. No overt abnormalities were found in the *Nha2* cKO mice. Data in a-c are expressed as the means±SD (*n*=3). (**d**-**f**) H&E staining of testis (d) and cauda epididymis (e) and morphology of spermatozoa obtained form cauda epididymidis (f). Scale bar in d and e, 100 μm; Scale bar in f, 10 μm. (**g**-**l**) Testicular weight (g), epididymal weight (h) and sperm count (i) were unchanged. No overt morphological abnormalities were observed in the testis (j) and cauda epididymis (k) of *Nha1/2* dKO mice. No structural abnormalities of *Nha1/2* dKO spermatozoa were observed (l). Data in g-i are expressed as the means±SD (*n*=3). Scale bar in j and k, 100 μm; Scale bar in l, 10 μm.

**Figure S3** *Nha2* cKO male mice are subfertile. (**a**) Pregnancy rate was calculated as the ratio of the number of females with pregnancy to the number of females with successful mating. (**b**) When calculating average litter size, only the females that generated pups were included. (**c**) Percentage motility of *Nha2* cKO and wild-type spermatozoa after release from the cauda epididymis (*n*=3). (**d**) cAMP content of spermatozoa was measured by using RIA (*n*=3). (**e**) The protein level of sAC_fl_ (normalized and plotted against β-tubulin) in *Nha2* dKO and wild-type spermatozoa (*n*=3). Data in a-e are expressed as the means±SD, **p*<0.05.

**Figure S4** Relative mRNA levels of *Nha1* and *Nha2* in cKO sperm samples. The mRNA expression of *Nha2* in *Nha1* cKO sperm (**a**) and the level of *Nha1* after *Nha2* deletion (**b**). *Gapdh* served as the internal control gene. The data are expressed as the mean±SD. **p*<0.05.
