# Supplementary figures and images for: Sodium–hydrogen exchanger NHA1 and NHA2 control sperm motility and male fertility
Source: Cell Death Dis. 2016 Mar 24;7(3):e2152–. doi: 10.1038/cddis.2016.65 (PMC4823964; doi:10.1038/cddis.2016.65)

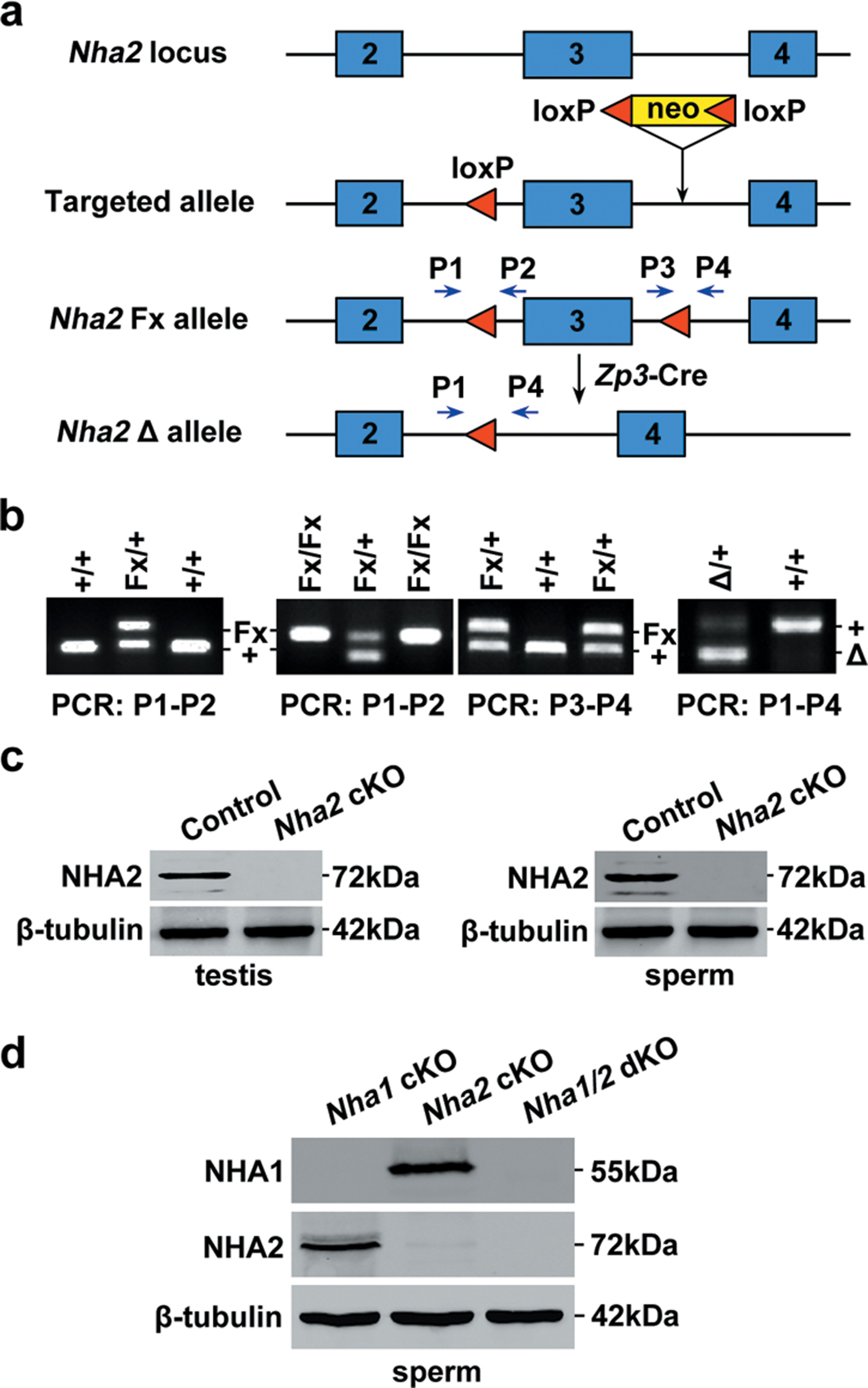

Supplement: Supplementary Figure 1 [file cddis201665x2.tif]

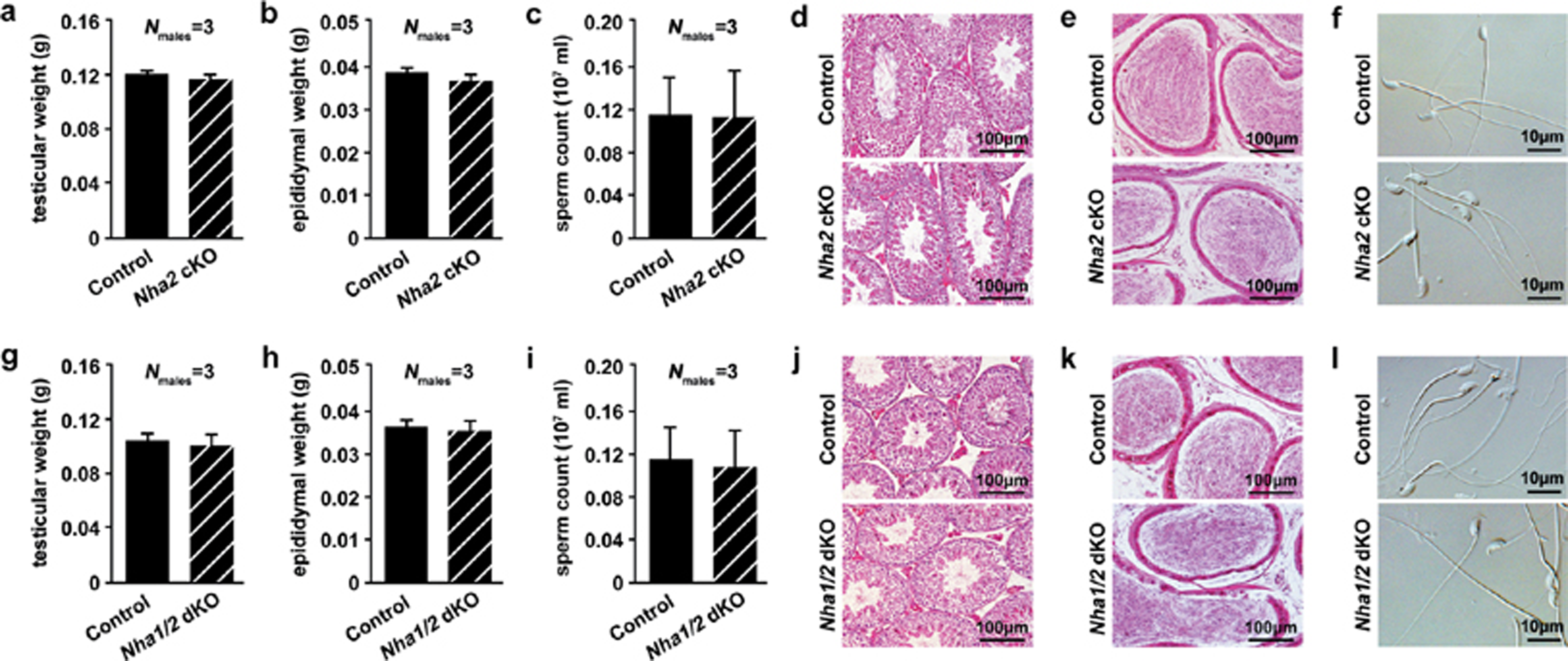

Supplement: Supplementary Figure 2 [file cddis201665x3.tif]

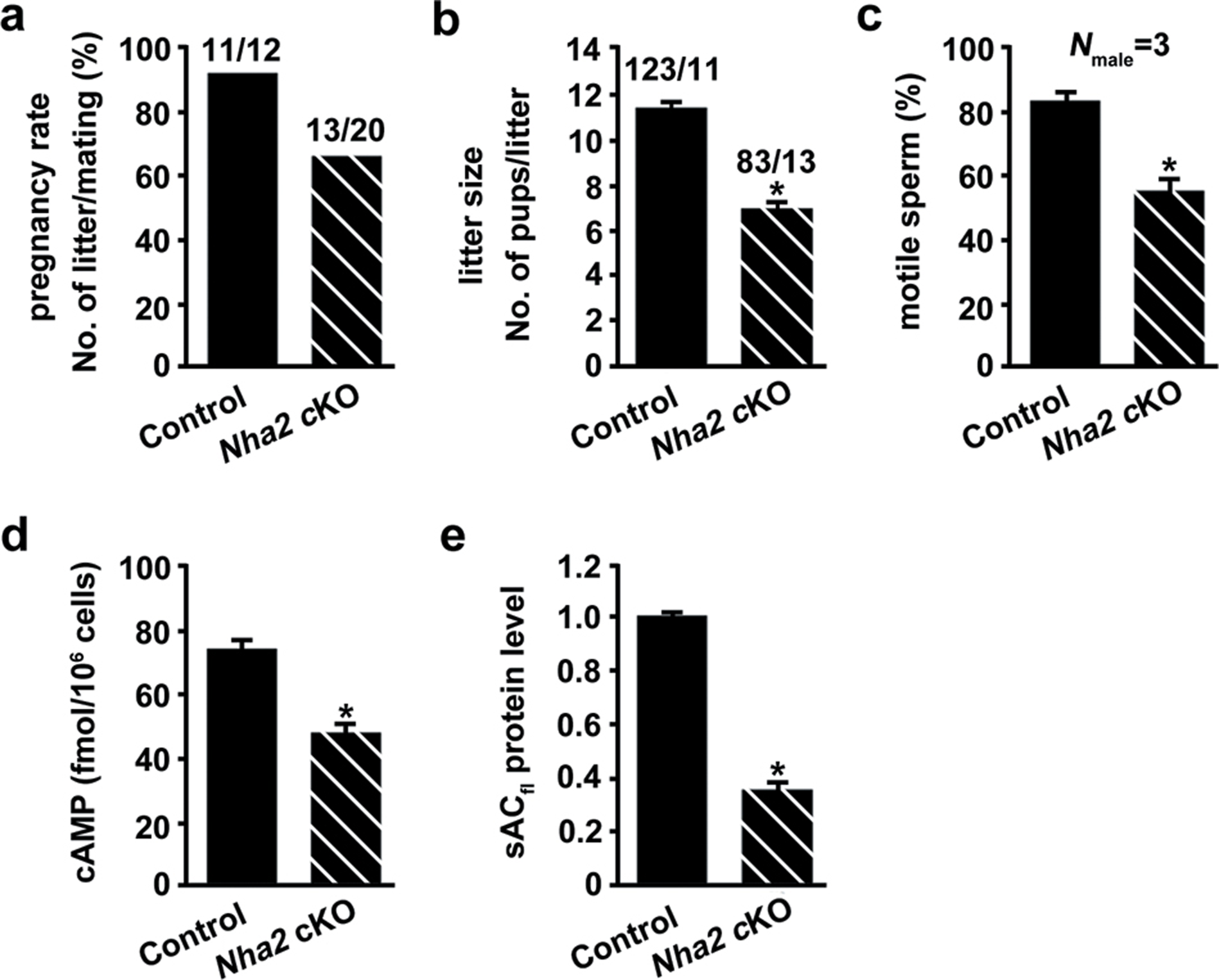

Supplement: Supplementary Figure 3 [file cddis201665x4.tif]

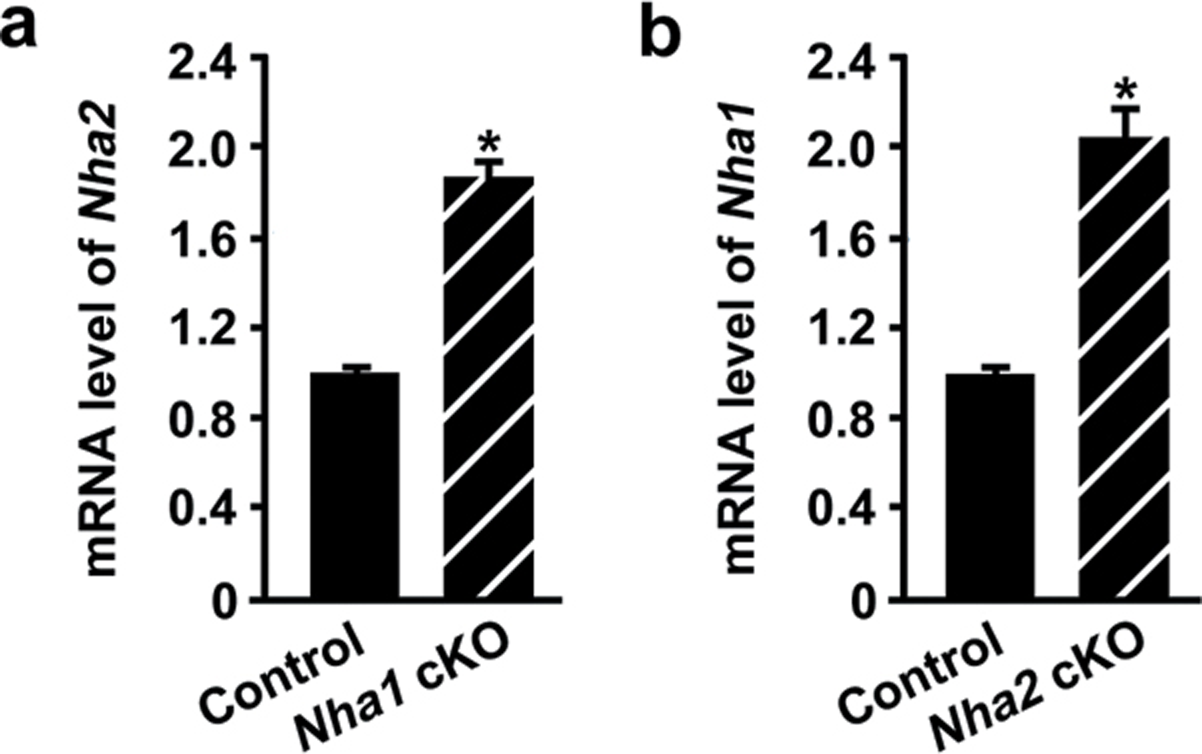

Supplement: Supplementary Figure 4 [file cddis201665x5.tif]
